# Nonlinear responses to temperature and precipitation shape the distribution of *Aedes sierrensis* in North America

**DOI:** 10.64898/2026.07.28.741332

**Published:** 2026-07-30

**Authors:** Erin Mordecai

## Abstract

Understanding the ecological determinants of species ranges is a central goal of ecology. Novel tools like global datasets and machine learning models allow us to describe species ranges with increasing scope and accuracy, and to develop and test ecological hypotheses about their determinants. Here, I focus on the widespread nuisance mosquito and dog heartworm vector *Aedes sierrensis* , a tree hole-breeding mosquito that is widespread and abundance within its native range in western North America, and develop species distribution models (SDMs) to characterize the species range and its environmental determinants. I find that the species range is highly predictable from long-term average bioclimatic variables. Temperature and precipitation were the primary determinants: suitability was highest at wet-season average temperatures of 0 – 10°C, minimum temperatures of −5 – 5°C, and summer temperatures of 8 – 22°C in environments with adequate seasonal rainfall concentrated in the winter. After accounting for climate, land cover variables showed minimal importance for prediction, but suitability was higher in forests and outside of urban areas. The results are consistent with ecological knowledge of the *Ae. sierrensis* life cycle from field observations and previous laboratory experiments, suggesting that individual physiological constraints scale up to determine species distributional limits.

## Full Text Availability

The license terms selected by the author(s) for this preprint version do not permit archiving in PMC. The full text is available from the preprint server.

